# xCT as a potential marker for neuroendocrine cells in high-risk prostate cancer and the relation to AL122023.1-miR-26a/30d/30e axis

**DOI:** 10.1371/journal.pone.0318213

**Published:** 2025-01-27

**Authors:** Elena D. Wilhelm, Jaroslaw T. Dankert, Marc Wiesehöfer, Sven Wach, Mathias Wagner, Martin Spahn, Marianna Kruithof-de Julio, Gunther Wennemuth

**Affiliations:** 1 Department of Anatomy, University Hospital Essen, Essen, Germany; 2 Department of Urology and Pediatric Urology, University Hospital Erlangen, Erlangen, Germany; 3 Department of General and Special Pathology, University Hospital Saarland, Homburg, Germany; 4 Department of Urology, Lindenhofspital Bern, Bern, Switzerland; 5 Department of Urology, University Hospital Essen, Essen, Germany; 6 Department for BioMedical Research, Urology Research Laboratory, University of Bern, Bern, Switzerland; 7 Department of Urology, Inselspital, University Hospital Bern, Bern, Switzerland; University of Mississippi Medical Center, UNITED STATES OF AMERICA

## Abstract

Prostate cancer is the second most common type of cancer in male worldwide. Stromal-epithelial interaction is thought to have a major impact on cancer development and progression. Previous studies have shown that interaction via soluble factors lead to a reduction in the expression of xCT and AL122023.1 in the prostate carcinoma cell line LNCaP after seven days of co-culture with primary stromal p21 cells. In this study, we validated the repression of *xCT* and *AL122023*.*1* at RNA level using quantitative real-time PCR and at protein level by Western Blotting. Furthermore, xCT is known to be a putative target for miRNAs miR-26a, miR-30d and miR-30e, which in turn potentially interact with AL122023.1. The lncRNA-miRNA-interaction was verified by luciferase reporter assays. However, miR-26a/-30d/-30e did not inhibit xCT expression at protein level. Nevertheless, indirect inhibitory effect of AL122023.1 on the xCT expression could be shown. Moreover, immunostaining revealed precise xCT expression in neuroendocrine cells, ranging from fetal, healthy juvenile, and adult prostate tissue to benign prostatic hyperplasia and finally advanced prostate cancer. This study explores the relevance and function of xCT and AL122023.1 in the prostate and exposes xCT as a potential marker or therapeutic target in high-risk prostate cancer.

## Introduction

Most commonly, prostate adenocarcinoma is a malignant transformation of glandular epithelial cells. According to Globocan 2020, prostate cancer (PCa) is the second most common cancer in men worldwide [1]. Although there are numerous treatment options with curative intent for the localized, organ-confined stage, more than 370,000 people still suffer from death due to PCa each year. Despite these treatments, there is still much to learn about this heterogeneous cancer type, particularly regarding its development and progression.

From a histological perspective, the prostate consists of epithelial and stromal cells that interact closely. Prostatic glands are mainly composed of luminal secreting epithelial cells, which pinch off secretory granules either apically or via exocytosis into the glandular lumen (apo- or merocrine) [2]. The glandular epithelium also contains basal stem cells, which are crucial for epithelial renewal, and few neuroendocrine cells (NE cells), which secrete various neuropeptides, such as chromogranin A, serotonin, and neuron-specific enolase [3]. In addition, NE cells are distinguished from other cell types by the absence of the androgen receptor [4,5]. Regarding the histogenesis, Szczyrba and colleagues demonstrated that neuroendocrine cells of the prostate originate from the neural crest [6]. Currently, however, the exact function of prostatic NE cells is not well understood, but they are thought to have a paracrine influence on the growth and differentiation of surrounding prostate cells. The stroma instead is mainly composed of smooth muscle cells and fibroblasts surrounding the prostate glands [7]. Smooth muscle cells contribute to the expulsion of prostatic secretions from the glands through their contractile activity. Besides, fibroblasts synthesize components of the extracellular matrix, such as glycoproteins, proteoglycans, or collagens to form a structural network. Vessels, immune cells, and nerve fibers are also present in the stroma. Further research on the interaction of epithelial and stromal cells will provide an important basis for a deeper understanding of the development and progression of PCa.

We previously published the mRNA expression profile of LNCaP prostate carcinoma cells and stromal p21 cells after co-culture, focusing on soluble factors, and highlighted *GALNT14* as a prominently induced gene [8]. Here, we furthermore identified and investigated *xCT* (*SLC7A11*) and *AL122023*.*1* as co-culture specific, repressed genes in LNCaP cells after paracrine interaction with p21 cells for seven days.

AL122023.1 is a long non-coding RNA (lncRNA) whose function is yet unknown. Besides protein-coding genes, the genome contains numerous non-coding RNAs, classified into short (smaller than 200 nucleotides) and long (between 200–1000 nucleotides) non-coding RNAs [9,10]. Among others, lncRNAs are considered to function as sponges or scavengers to sequester microRNAs (miRNAs). MiRNAs themselves are about 18–23 nucleotides long, single-stranded, non-coding RNA molecules. The miRNA seed sequence is crucial for binding to their target sequence, usually located within the 3’-untranslated region (3’UTR) of mRNAs. This miRNA-mRNA interaction typically inhibits target mRNA translation [11,12]. Consequently, if miRNAs are captured by lncRNAs, they are no longer able to post-transcriptionally regulate their target mRNAs which could thus function either oncogenic or tumor suppressive [13]. Especially, lncRNAs are involved in transcriptional, post-transcriptional and translational regulation and are relevant regarding tumor biology. Recent publications suggest that lncRNAs play a role in the development of resistance to therapeutic agents in PCa [14]. In addition, using the bioinformatics prediction tools DIANA and TargetScan, an indirect link of AL122023.1 to xCT via miR-26a, miR-30d and miR-30e is assumed. xCT, also referred to as SLC7A11 (Solute Carrier Family 7 Member 11), together with SLC3A2 (synonyms: 4F2, CD98) forms a membrane-bound, sodium-independent amino acid antiporter [15]. This heterodimer (Xc^-^-system) exchanges cytosolic glutamate for extracellular cystine [16] to protect cells against oxidative stress and to maintain the redox balance [17]. Furthermore, xCT is functionally involved in the inhibition of ferroptosis [18]. This iron-dependent cell death might have a clinical relevance in terms of new therapeutic approaches in cancer. This study characterizes xCT in human prostate tissue of different developmental stages as well as pathology and elucidates the relation to AL122023.1, miR-26a and miR-30d/e.

## Materials and methods

### Cell lines and transfections

HEK293T cells (*Human Embryonic Kidney* 293 cells; RRID:CVCL_0063; American Type Culture Collection ATCC/LGC Standards GmbH, Wesel, Germany) were grown in DMEM (*Dulbecco’s Modified Eagle Medium*; Thermo Fisher Scientific, Schwerte, Germany) supplemented with 10% heat-inactivated fetal bovine serum (Life Technologies (Gibco), Thermo Fisher Scientific, Oberhausen, Germany), 100 U/ml penicillin and 100 μg/ml streptomycin. LNCaP cells (*Lymph Node Carcinoma of Prostate* cells; RRID:CVCL_0395; Sigma-Aldrich, Hamburg, Germany) were cultivated in RPMI 1640 medium (*Roswell Park Memorial Institute* 1640; Thermo Fisher Scientific, Oberhausen, Germany) with same supplements and additionally 1 mM pyruvate (Thermo Fisher Scientific, Schwerte, Germany). Cells were last authenticated by ATCC, performing STR Profiling following ISO 9001:2008 and ISO/IEC 17025:2005 quality standards. Exclusion of Mycoplasma infection was tested as previously described [8]. Cultivation of primary p21 cells as well as their co-cultivation with LNCaP cells was performed as described priorly [8].

In general, 5x10^5^ LNCaP cells or 6x10^5^ HEK293T cells were seeded into 6-well plates, transfected with 2 μg expression plasmid DNA using jetPRIME transfection reagent (Polyplus transfection, Sélestat, France) on the following day to finally isolate RNA or protein after further 48 h. As transfection control, GFP (transfection with pAcGFP-C1 expression plasmid) was detected by microscope *Eclipse Ni* (Nikon, Amsterdam. Netherlands).

### Human prostate specimens

Same samples were used as described previously [8]. The Institute of General and Special Pathology at Saarland University Hospital in Homburg provided fetal tissue (n = 2), normal tissue of different age (n = 9), BPH (n = 5) and rhabdomyosarcoma (n = 1) (ethics approval University of Duisburg-Essen 18-7959-BO). Additional fetal tissue (n = 6) was obtained from Dr. Laurence Baskin at the Department of Urology, University of California, San Francisco (UCSF) (local ethics approval plus inclusion in 18-7959-BO). Prostate carcinoma tissue was provided by the following collaborations: Tissue biobank of the Comprehensive Cancer Center (CCC-ER EMN) of the University Hospital Erlangen in cooperation with Prof. Dr. Helge Taubert and Dr. Sven Wach (project no. 2019–112; n = 11 cryopreserved); Department of Urology at the Community Hospital Karlsruhe or EMPaCT tumor bank (European Multicenter Prostate Cancer Clinical and Translational Research Group) in cooperation with Prof. Dr. Martin Spahn, PD Dr. phil. Marianna Kruithof-de Julio and Dr. phil. Eugenio Zoni (n = 203 of N = 180 paraffin-embedded) (ethics approval KEK Bern No. 128/2015) [19]. Tissue microarrays (TMAs) were generated by multiple tumor samples derived from the index lesion including more differentiated areas of each tumor as well as matched lymph node metastasis from previously untreated patients. The expression of several tumor relevant genes (e.g. AR, PTEN, p53, MLH1, CD44, ALDH1, chromogranin A, and synaptophysin) and the TMPRSS2-ERG gene fusion was also analyzed on TMAs [20,21]. Pathological tissue was reviewed by local pathologists at the respective sites.

### RNA isolation, cDNA synthesis and quantitative real-time PCR

Total RNA was isolated from cell lines using the miRNeasy Mini Kit (Qiagen, Hilden, Germany) according to the manufacturer’s instruction. The BioPhotometer (Eppendorf, Hamburg, Germany) was used to determine the RNA concentration and quality.

cDNA synthesis from 1 μg total RNA and analysis of the relative gene expression was realized as previously described [8]. Besides, miRNAs (10 ng) were transcribed into cDNA using miRCURY LNA RT Kit (Qiagen, Hilden, Germany) according to manufacturer’s instruction. QRT- PCR was performed using the miRCURY LNA SYBR® Green PCR Kit (Qiagen, Hilden, Germany) according to the manufacturer’s protocol and the real-time PCR detection system qTower^3^G (Analytic Jena, Jena, Germany). To quantify the target gene expression, we used 5S rRNA as the reference gene for miRNAs, and GAPDH along with HPRT1 as reference genes for mRNAs, using the ΔΔCt method. Duplicates and Non Template Controls (NTC) were included in every experiment. The thermal cycling conditions for miRNAs were as follows: 95°C for 2 min followed by 40 cycles of 95°C for 10 sec and 56°C for 60 sec. To ensure purity and specificity of PCR, a melting curve analysis was performed. S1 Table lists all oligonucleotide sequences for mRNA and miRNA detection.

### Non-radioactive Northern blot

At least 10 μg total RNA were separated on Mini-PROTEAN^®^ TBE-Urea Precast gels or on 12% denaturing urea PAGE (SequaGel-UreaGel System; National Diagnostics, Beutelsbach, Germany). To ensure a comparable loading, gels were immersed in GelRed (1:20000 in TBE) and RNA detected by UV light with the ChemiDoc Touch Imaging System (Bio-Rad, Hercules, CA USA). RNA was then transferred to the nylon membrane Amersham Hybond-N+ (GE Healthcare Life Science, Freiburg, Germany) in a semi-dry transfer cell (BioRad, Munich, Germany; 30 min at 15 V) and chemically cross-linked with the soluble carbodiimide, EDC, for 60 min at 55°C. Hybridization of blots with 5’biotin-labeled antisense probes was performed at 50°C overnight and membranes were then washed twice with 5X SSC/ 0.1% SDS and 1X SSC/ 0.1% SDS for 15 min at 50°C. For detection of hybridized probes, HRP conjugated Streptavidin (R&D Systems, Minneapolis, MN USA, 1:1000) was incubated for 1 h and detected by the Clarity^TM^ Western ECL Substrate according to the manufacturer’s specifications. Target miRNAs were finally visualized by the ChemiDoc Touch Imaging System (Bio-Rad, Hercules, CA USA). S2 Table contains the probe sequences.

### Target prediction

TargetScan (release 8.0; http://www.targetscan.org/) was used for miRNA target prediction. Databases DIANA (release lncBase v.3; https://diana.e-ce.uth.gr/lncbasev3/interactions) and Starbase (release v2.0; https://starbase.sysu.edu.cn) helped to predict lncRNA-miRNA-interaction.

### Plasmids

All oligonucleotide sequences were amplified from human genomic DNA. MiR-26a (nucleotides 37969254–37969610 of chromosome 3), miR-30d (nucleotides 134804658–134805143 of chromosome 8) and miR-30e (nucleotides 40754221–40754542 of chromosome 1) were inserted into the pSG5 expression vector (Agilent technologies, Ratingen, Germany). AL122023.1 (amplicon size: 530 bp, nucleotides 93334528–93335057 of forward strand on chromosome 14 (GRCh38), Ensembl number: ENSG00000278396) was inserted into the pcDNA3.1(+) expression vector (Thermo Fisher Scientific, Oberhausen, Germany). QRT-PCR and Northern Blotting verified overexpression in HEK293T or LNCaP cells (S4 Fig).

For luciferase reporter assays, the AL122023.1 sequence was inserted into the modified pMIR-RNL-TK reporter vector (Thermo Fisher Scientific, Oberhausen, Germany) as described elsewhere [22]. The reporter plasmid expresses the firefly luciferase mRNA including the sequence of interest and constitutively expresses the renilla luciferase as an internal normalization control. Mutagenesis of the predicted binding sites for miRNAs were performed by site directed mutagenesis and with sequence specific primers. S3 Table depicts all oligonucleotide sequences for molecular cloning.

### Dual-Luciferase reporter assay

To analyze the lncRNA-miRNA-interaction, 2x10^5^ HEK293T cells were seeded into a 24-well plate. Cells were then transfected with 0.2 μg pMIR-RNL-TK reporter plasmid (containing lncRNA) and 0.8 μg pSG5 effector plasmid (containing miRNA) using JetPrime (Polyplus transfection, Illkirch, France) on the following day. After an incubation for 48 h, cells were prepared for the dual luciferase reporter assay system (Promega, Mannheim, Germany) according to the manufacturer’s instruction. Detection of luminescence was exhibit using the luminometer Lucetta (Lonza, Basel, Schweiz). To quantify the relative luciferase activity, the determined light emission of the firefly luciferase was normalized with that of the constitutively expressed renilla luciferase. Values to be compared were finally correlated.

### Protein extraction and Western Blotting

5x10^5^ LNCaP cells or 6x10^5^ HEK293T cells were seeded into a 6-well plate and after 48 h, transfected with 2 μg expression plasmid DNA using jetPRIME transfection reagent (Polyplus transfection, Illkirch, France). After two further days, protein lysates were extracted with 90 μl RIPA buffer (5 M NaCl, 0,5 M EDTA (pH 8,0), 1 M Tris (pH 8,0), 10% Natriumdesoxycholat, 10% SDS plus protease/phosphatase inhibitor). Protein lysates were supplemented with denaturating gel loading buffer (4X Laemmli buffer: 8% SDS, 20% 3-Mercapto-1,2-propandiol, 40% Glycerol, 0,008% Bromphenolblau, 0,25 M Tris HCl) and separated by electrophoresis on 8–16% *Mini- PROTEAN TGX Precast* gels or on 12% gels using the *TGX Stain-free FastCast Acrylamide Kit* (Bio-Rad, Hercules, CA USA). Separated proteins were transferred by semi-dry blotting on the *Trans-Blot Turbo* PVDF membrane using the *Trans-Blot Turbo Transfer System* (Bio-Rad, Hercules, CA USA). The membrane was blocked for 45 min under gentle shaking, using either BSA or skim milk dissolved in 1x TBST (2 mM Tris HCl, 13.7 mM NaCl, 0,1% Tween 20), depending on the primary antibody. For immune detection, the following primary antibodies were were diluted in their respective blocking solutions and incubated over night: Rabbit anti-SLC7A11/xCT (12691S, clone D2M7A, Cell Signaling, Leiden, Netherlands; 1:1000 in 5% BSA) and rabbit anti-GAPDH (AB181602, Abcam, Cambridge, UK; 1:1000 in 2% skim milk). A HRP-conjugated goat anti-rabbit IgG (111-035-144, Jackson Immuno-Research, PA, USA; 1:10.000 in corresponding blocking solution) was incubated for 1 h. Between antibody treatments, the membrane was washed three times for 15 min each with 1x TBST. Finally, the Clarity Western ECL Substrate was used to detect proteins according to the manufacturer’s specifications and detected by the ChemiDoc Touch Imaging System in auto-detect mode (Bio-Rad, Hercules, CA USA).

### Immunohistochemical and immunofluorescence staining

Immunohistochemical and immunofluorescence staining was performed as previously described in detail [8]. Following primary antibodies were used: rabbit anti-SLC7A11/xCT (5 μg, PA-16893, Thermo Fisher Scientific, Oberhausen, Germany) and mouse anti-chromogranin A (1:100, ab715, Abcam, Cambridge, UK). A negative isotype control (IgG fraction of non-immunized rabbits or mouse anti-rat-CEACAM1 (IgG κ)) and a positive tissue control complemented each staining (S1 Fig).

Immunohistochemical evaluation of xCT expression was performed separately for glandular epithelium and tumor-associated stroma. In the glandular epithelium, xCT expression was assessed by determining the presence or absence of xCT-positive cells and correlated with the Gleason score. In the tumor-associated stroma, xCT expression was categorized into three levels based on the overall impression of staining: weak, moderate, and strong staining and also analyzed in relation to the Gleason score. Immunohistochemical stainings were evaluated subjectively without quantification due to the heterogeneous distribution and diffuse expression patterns.

### Statistical analyses

Quantification of Western Blots was performed by ImageJ 1.52a (National Institute of Health, Bethesda, MD, USA). Data of qRT-PCR and luciferase reporter assays were analyzed and visualized using SigmaPlot 13 (Systat, Erkrath, Germany) or GraphPad Prism 9 (GraphPad Software, San Diego, CA USA). The number of biological replicates (n) is specified in the figure legends for each experiment. Biological replicates refer to independent samples, such as separate cell culture experiments, Western Blot analyses, qRT-PCR or distinct tissue samples. For experiments involving technical replicates (e.g., duplicate measurements from the same sample), this is also indicated in the figure legends (e.g., “n = 4, measured in duplicates”). Differences between two sets of data were statistically evaluated with Student’s unpaired, two-tailed t-test or Mann-Whitney U-test. Probability values (p-values) <0.05 were considered as significant and indicated with *, ** p<0.01, *** p<0.001. All results are represented as mean ± standard deviation from at least three independent replicates. To perform prostate cancer/normal differential expression analysis, the database GEPIA (Gene Expression Profiling Interactive Analysis) was used with default adjustments [23].

## Results

### xCT and AL122023.1 as repressed genes after stromal-epithelial interaction

**Fig 1:**
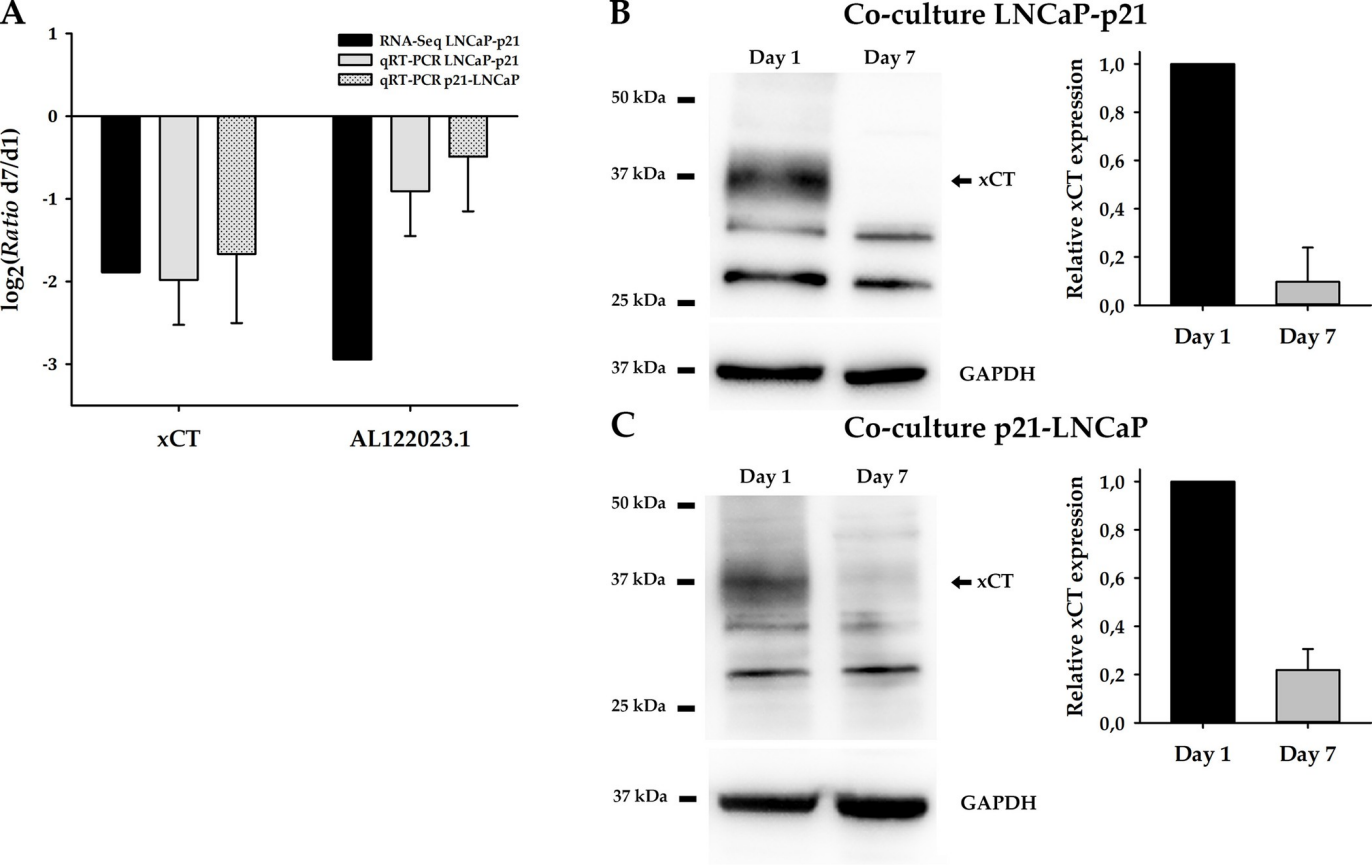
Analysis of *xCT* and *AL122023*.*1* expression in LNCaP cells after interaction with stromal p21 cells via soluble factors for seven days. (**A**) RNA sequencing data (black bar, RNA seq LNCaP-p21) are validated in three independent co-culture experiments by qRT-PCR (grey bar, co-culture LNCaP-p21). Additionally, repression of *xCT* and *AL122023*.*1* is also verified in the reciprocal co-culture (grey dotted bar, co-culture p21-LNCaP). HPRT1 and GAPDH both served as reference genes. Binary logarithm with standard deviation is displayed (n = 3). (**B**) (**C**) xCT (35 kDa) expression change in LNCaP cells is also confirmed on protein level by Western Blotting (n = 4) for both co-culture systems. GAPDH (36 kDa) served as loading control. d1, day 1; d7, day 7; 25 kDa, 37 kDa and 50 kDa depict the protein size marker.

In order to investigate the expression profiles of LNCaP cells after stromal-epithelial interaction via soluble factors, we previously performed RNA sequencing of LNCaP cells from co-culture experiments with stromal p21 cells for one and seven days [8]. *xCT* and *AL122023*.*1* were identified as the most profoundly repressed genes over time and were therefore chosen for further investigation. To verify the sequencing results, we performed three independent co-culture experiments followed by quantitative real-time PCR (qRT-PCR) (Fig 1A). In these experiments, LNCaP cells are cultured within a hanging cell culture insert, while p21 cells are placed in the well of a culture plate to create a system of stromal-epithelial interaction without direct contact. To take into account that soluble factors might be released in a specific orientation (apical or basal), we also performed the co-cultures in reverse orientation (n = 3). Again, qRT-PCR analyses confirm the repression of *xCT* and *AL122023*.*1* after co-cultivation. Additionally, we analyzed the expression changes from day 1 to day 7 in LNCaP monocultures as control for both experimental setups. Although an effect on xCT expression was also found in some cases, the results fluctuated greatly and were either constant or increased (S3 Fig). Since the lncRNA *AL122023*.*1* is not translated into a protein, the qRT-PCR analysis provides the final confirmation of the gene expression change. For the amino acid antiporter xCT, repression was further validated at protein level by Western Blot analyses (n = 3). After seven days of both co-culture setups (LNCaP-p21; p21-LNCaP), expression is reduced by 90,3% (LNCaP-p21) and 78% (p21-LNCaP) on average compared to day 1 (Fig 1B and 1C). To control the functionality of the antibody, xCT was additionally examined after overexpression in cells by Western Blotting (S3 Fig).

### AL122023.1-miR-26a/30d/30e axis

**Fig 2:**
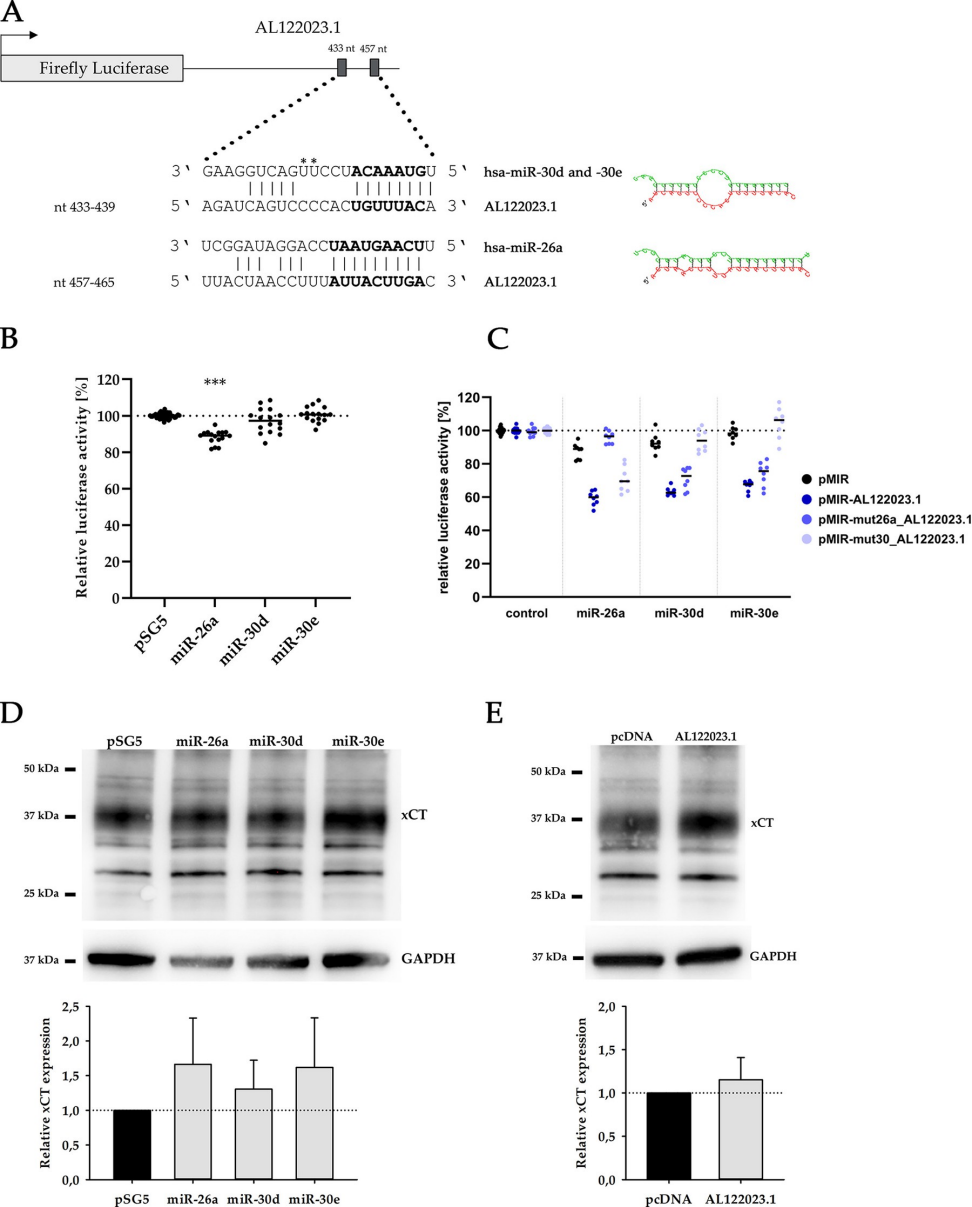
Mir-26a, miR-30d and miR-30e interact with the lncRNA AL122023.1. (**A**) Schematic illustration of predicted miRNA binding sites within the AL122023.1 sequence. RNAhybrid was used to determine the lowest free energy for hybridization of two RNA molecules (AL122023.1 and miR-30d/e: -20.4 kcal/mol, AL122023.1 and miR-26a 20.7 kcal/mol) and to generate an alternative depiction (right site). *, CC for miR-30d, apart from that identical. The complementary sequences corresponding to the seed sequences were removed by truncation of the AL122023.1 sequence. (**B**) Luciferase reporter assays were performed to investigate the lncRNA-miRNA-interaction. Control transfections of reporter plasmid (pMIR) with empty effector plasmid (pSG5) were set to 100% (illustrated by dashed line). Only miR-26a shows a significant effect on the reporter plasmid. ***, p<0.001. (**C**) MiR-26a, -30d, and -30e cause a significant reduction in luciferase activity of approximately 30% after interaction with AL122023.1 (pMIR-AL122023.1, dark blue) (n = 4, measured in duplicates). This effect can be reversed when miRNA binding sites are inaccessible (pMIR-mut26a_AL122023.1, mid blue; pMIR-mut30_AL122023.1, light blue) (**D-E**) Overexpression of miR-26a, -30d, -30e and AL122023.1 in LNCaP cells cause an induction of xCT protein amount. GAPDH (36 kDa) served as loading control. 25 kDa, 37 kDa and 50 kDa depict the protein size marker.

Using the databases DIANA and Starbase, miR-26a-5p, miR-30d-5p, miR-30e-5p were identified as putative interaction partners of *AL122023*.*1*. To test, if the identified miRNAs are able to interact with *AL122023*.*1*, luciferase reporter gene constructs were used. For this purpose, the entire sequence of *AL122023*.*1* (530 bp) was cloned downstream of the coding region of the firefly luciferase in the pMIR reporter plasmid to set the reporter gene under the regulative control of *AL122023*.*1*, now acting as a 3’UTR element. Fig 2A illustrates the predicted binding sites and the seed sequences of miR-26a and miR-30d/e, respectively. All miRNAs have one potential binding site in AL122023.1.

Reporter gene plasmids (pMIR, pMIR-AL122023.1, pMIR-mut_AL122023.1) were co-transfected with miRNA expression vectors (pSG5) in HEK293T cells. Interestingly, miR-26a shows a negative regulatory effect even in the absence of the AL122023.1 sequence where it causes a reduction of about 13% in reporter gene activity (p≤0.001), whereas miR-30d and miR-30e have no regulative effect on the reporter gene activity in the absence of the AL122023.1 sequence (Fig 2B). The presence of the AL122023.1 sequence, acting as a 3’UTR element, makes the reporter gene susceptible for a miRNA-mediated negative regulation. The luciferase activity decreases by an average of 28% (p≤0.001) for miR-26a, 29% (p≤0.001) for miR-30d and 31% (p≤0.001) for miR-30e (Fig 2C).

To verify the specificity of this regulation, the complementary sequences corresponding to the seed sequence, crucial for miRNA binding, were mutated via nucleotide exchange using site-directed mutagenesis (Fig 2C). This modification resulted in an increase in relative luciferase activity to the control levels when the corresponding miRNAs were cotransfected. Consequently, it can be concluded that miR-26a, miR-30d and miR-30e specifically bind to their individual predicted binding site within the lncRNA AL122023.1. The resulting pMIR-mut_AL122023.1 reporter gene construct is completely irresponsive for any miRNA-mediated gene regulation (Fig 2C). This confirms that the binding sites for the three miRNAs in the AL122023.1 lncRNA are functionally active and able to interact with miR-26a, miR-30d and miR-30e.

Furthermore, xCT was identified as a potential target gene of miR-26a, -30d and -30e using the database TargetScan, suggesting an indirect effect of AL122023.1 on xCT expression. To test this hypothesis, xCT expression was examined at protein level by Western Blotting after overexpression of each miRNAs separately and of AL122023.1 in LNCaP cells (Fig 2D and 2E). The expression of either miRNA leads to an increase in xCT protein levels. Overexpression of miR-26a results an average induction of xCT expression by 66%, miR-30d by 31%, and miR-30e by 62% (Fig 2D). Although we were able to confirm the functional interaction of miR-26a, miR-30d and miR-30e with the lncRNA *AL122023*.*1*, the proposed miRNA sequestering effect does not appear to be the functional basis of the observed xCT overexpression. In contrast, AL122023.1 overexpression increases xCT expression by an average of 15% (Fig 2E).

Despite the exact mode of xCT regulation remaining to be elucidated, we demonstrated a negative regulatory effect on xCT expression caused by co-cultivation of tumor cells with p21 primary stromal cells. This negative regulation could potentially render tumor cells more susceptible to cell death induced by oxidative stress. Consequently, we investigated the expression of xCT in primary tissues at various developmental and malignant stages.

### xCT expression in non-malignant prostate tissue

Regarding the localization and distribution in prostate tissue, xCT expression was investigated immunohistochemically in benign and malignant prostate tissues, as well as in healthy prostate tissues at different developmental stages. Fetal prostate tissue, which exhibits variable glandular appearances such as non-canalized glands, was first stained with the epithelial marker cytokeratin 7 to provide a reference staining (S1 Fig). To visualize muscle cells in the stroma, the type 3 intermediate filament desmin was additionally detected before examining xCT (S1 Fig).

**Fig 3:**
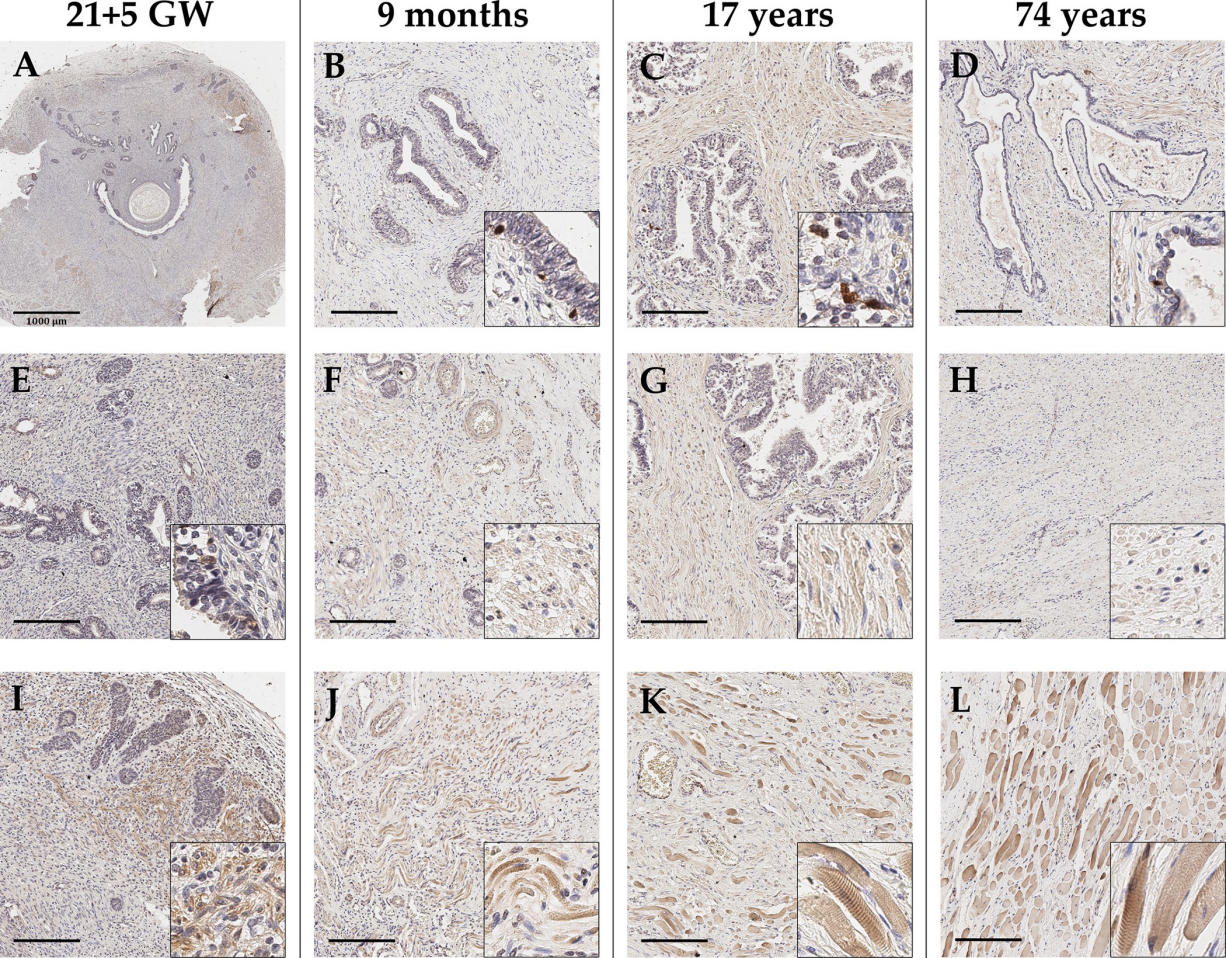
Representative immunohistochemical staining of healthy prostate tissue across different ages, ranging from fetal over juvenile to adult stages (column-wise) to detect xCT. (**A-E**) Glandular epithelium is weakly positive for xCT in fetal tissue, whereas postnatal tissue shows distinct strong xCT-positive cells. (**F-I**) In adjacent stroma a weak to moderate xCT expression is detected. (**J-L**) Present skeletal muscle consistently is strongly xCT-positive. In general, no age-related change can be observed. Scale bar, 200 μm.

In five out of eight samples, xCT staining is rather weak or barely present in epithelial cells (gestation week: 12; 14.5; 17.1; 18.2; 21.5, with the latter representatively depicted in Fig 3A, 3E and 3I). One sample exhibited a moderate staining of the glandular epithelium (gestation week 14.6). In two cases, distinct strongly xCT-positive cells are detected basally in the epithelium (gestation week: 18; 21.5). In the stroma, xCT-positive cells are mostly detected in marginal areas (Fig 3I).

To investigate an age-dependent expression of xCT, healthy juvenile and adult tissues were stained. Specimens from males of different ages (gestation week 21.5, 9 months, 17 years, and 74 years) are representatively demonstrated (Fig 3). Notably, single strongly xCT-positive cells are present in the glandular epithelium independent of the age (Fig 3B–3D). These cells are mostly located basally and are smaller in size compared to adjacent epithelial cells. In the stroma, certain cells weakly expressed xCT (Fig 3F–3H), though the specific cell type was not identified. Furthermore, present skeletal muscle as well as perikarya of neurons are markedly xCT-positive (Figs 3J–3L and S2). Finally, endothelial cells of some blood vessels show a weak xCT expression, with the *tunica media* being more xCT-positive in larger vessels. Overall, no age-related increase or decrease of xCT-positive cells in the epithelium or stroma can be detected.

### xCT expression in benign prostatic hyperplasia (BPH) and rhabdomyosarcoma of the prostate

BPH is a benign enlargement of the prostate gland whereas the rhabdomyosarcoma is a malignant neoplasm originating from muscle cells. Both were used to study xCT expression in pathologically altered tissue in addition to PCa.

**Fig 4:**
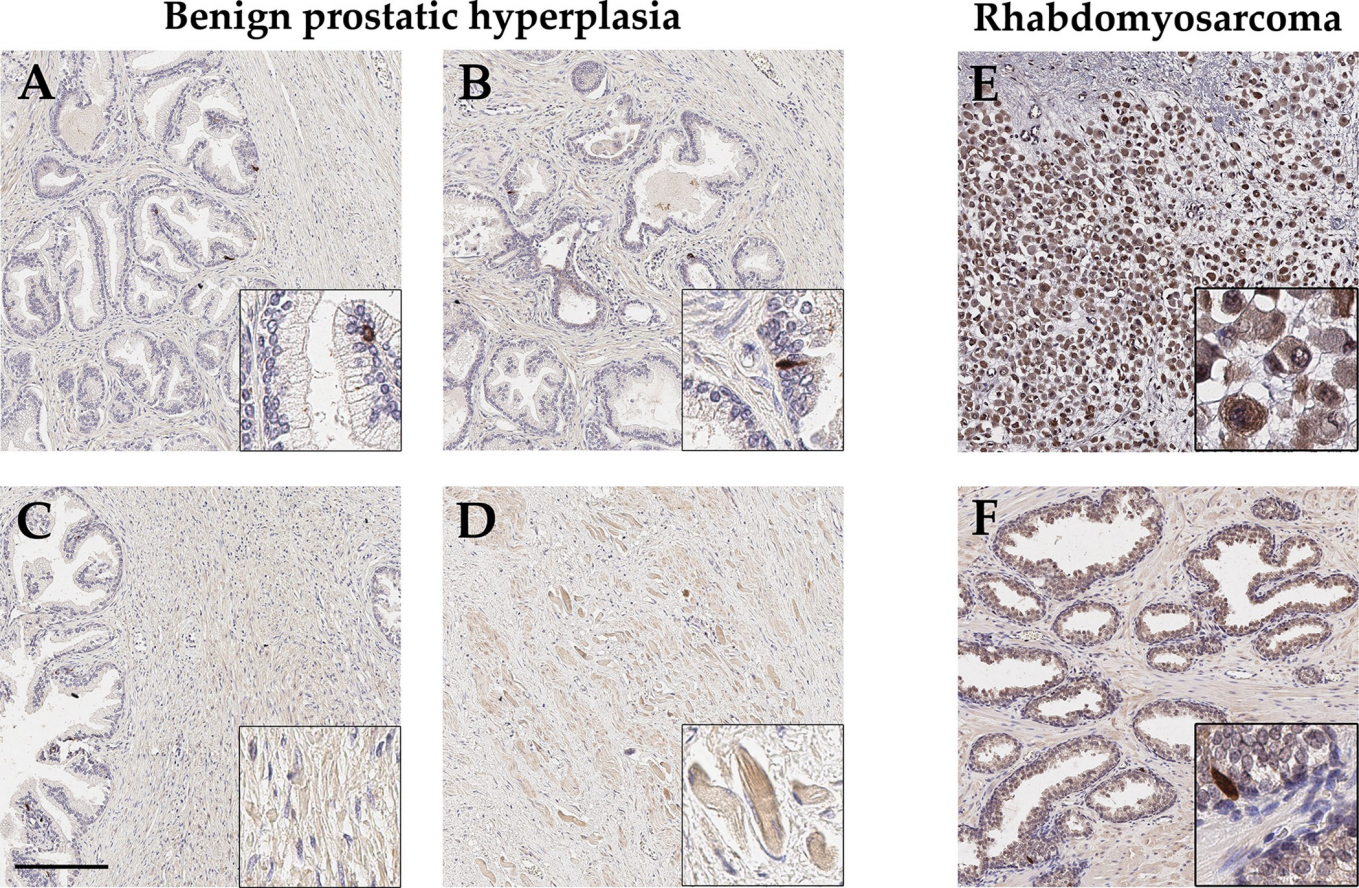
Representative immunohistochemical staining of benign prostatic hyperplasia (BPH) and rhabdomyosarcoma with anti-xCT antibodies. (**A-B**) In BPH, individual cells within the glandular epithelium show an intense xCT expression. (**C-D**) Certain stromal cells appear weakly xCT-positive, whereas skeletal muscle cells display a distinct xCT expression. (**E-F**) In rhabdomyosarcoma, tumor cells strongly express xCT, while adjacent normal tissue shows the typical staining of individual cells within the glandular epithelium. Scale bar, 200 μm.

In BPH tissue (n = 5; age: 60, 66, 70, 76, 86), the glandular epithelium is predominantly xCT-negative, with isolated strongly xCT-positive cells (Fig 4A and 4B). Generally, these cells are located basally and have a small cell size. The stroma shows a heterogeneous staining pattern, with only one specific cell type appearing xCT-positive, but irregularly distributed (Fig 4C). In skeletal muscle distant from glands and in perikarya of neurons, xCT is detectable, whereas the endothelium of small blood vessels is rather weakly xCT-positive (Figs 4D and S2). A more pronounced staining is visible only in larger vessels with a *tunica media*. In summary, xCT expression in BPH mirrors that in healthy juvenile and adult tissue.

To investigate the embryonal rhabdomyosarcoma (ERMS, n = 1) of the prostate of a 17-year-old male, different control stainings were first conducted and shown in a previously published study [8]. In that study, the marker protein desmin was visualized to detect the tumor cells. Additionally, staining with anti-CD68-antibodies was performed to distinguish tumor cells from macrophages. Furthermore, a cytokeratin 7 reference staining was applied to test whether intact glandular epithelium is present. In this study, we focus solely on the detection of xCT in the corresponding tissue. The tumor lacks intact glandular tissue but contains numerous desmin-positive cells [8], which also clearly express xCT (Fig 4E). Adjacent normal prostate tissue to the ERMS exhibits the typical staining of distinct cells within the glandular epithelium (Fig 4F).

### xCT expression in prostate carcinoma

**Fig 5:**
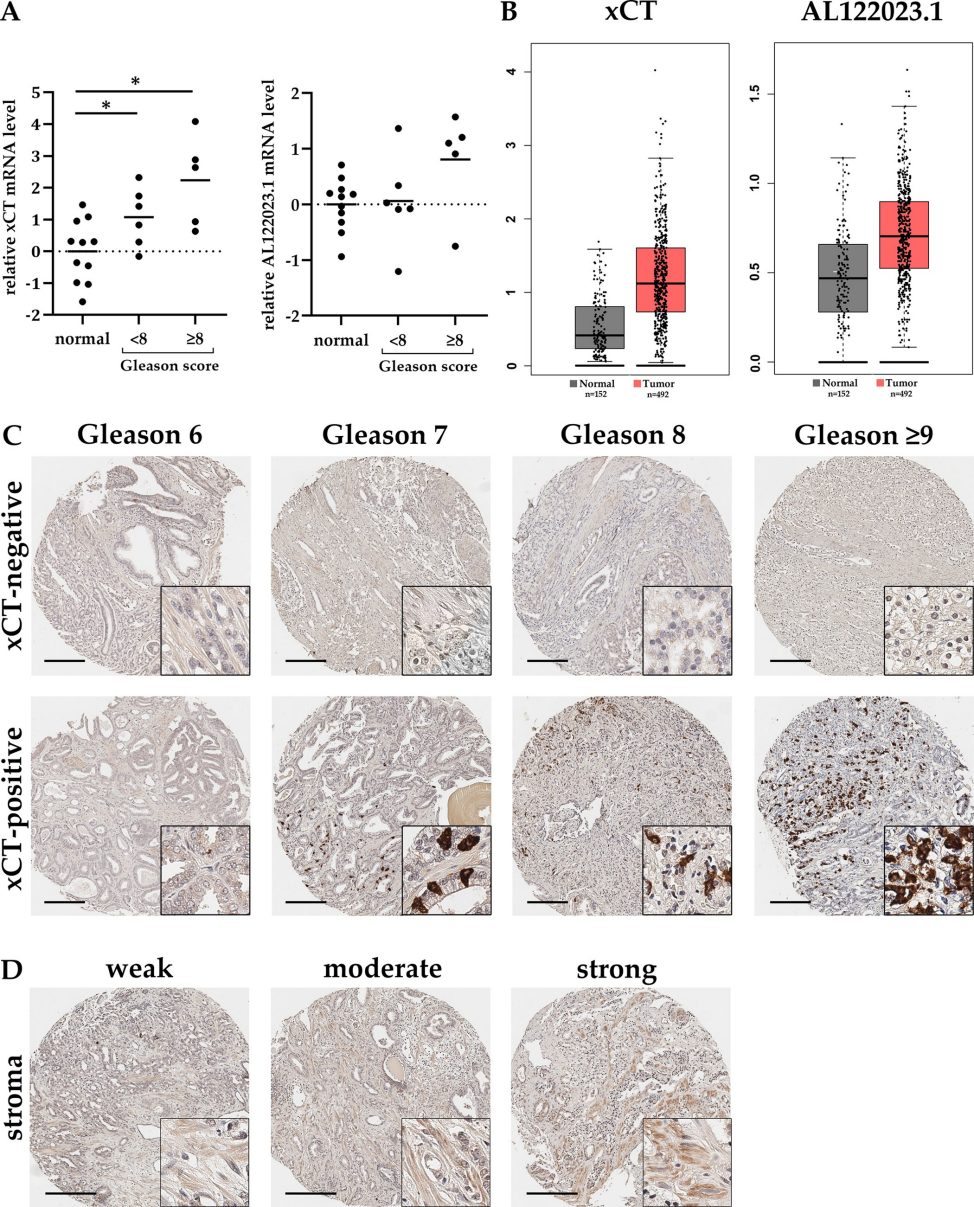
xCT expression in PCa samples with different Gleason score. (**A**) To determine the relative expression change (ratio) of xCT and AL122023.1, cryopreserved PCa samples with Gleason score <8 (n = 6) or ≥8 (n = 5) were compared to adjacent normal tissue (n = 11). QRT-PCR results reveal an induction of xCT and AL122023.1 expression with increasing Gleason score (unpaired t-test for normally distributed data; * p<0.05). Expression in normal tissue is shown as distribution around the mean. HPRT1 and GAPDH both served as reference genes. (**B**) Results from the GEPIA Database confirm the induced expression of *xCT* and *AL122023*.*1* in PCa (visually adjusted graph). (**C**) Tumor microarrays with 196 PCa biopsies with different Gleason scores were assessed after immunohistochemical staining with anti-xCT-antibodies. Two representative samples are shown per Gleason score (6, 7, 8, ≥9), distinguishing between xCT-negative (no detection of cells expressing xCT) and xCT-positive (detection of xCT expression in cells). The number of xCT-positive cells raises starting from Gleason score 7. (**D**) xCT expression was also investigated in associated stroma and samples were classified into absent, weak, moderate, or strong staining as shown in the representative images. Scale bar, 200 μm.

To examine the presence of xCT in PCa tissue, the overall expression at RNA level was determined by qRT-PCR. Six cryopreserved PCa samples with a Gleason score less than 8 (<GS8) and five with a Gleason score greater than or equal 8 (GS≥8) as well as their respective adjacent normal tissue were included in this study. For the comparative analysis between normal and PCa tissue, xCT expression in normal tissue was initially quantified as a distribution around the mean, and then compared to its expression in PCa tissues. While *xCT* expression in normal tissue varies greatly between repressed and induced, there is an average induction of *xCT* expression in PCa compared to normal tissue (*, p≤0.05) (Fig 5A). Additionally, there is a trend of increasing xCT expression with higher Gleason scores. Specifically, xCT expression doubles comparing samples <GS8 to those with ≥GS8 (average log_2_ ≈ 1 in <GS8 and log_2_ ≈ 2.2 in ≥GS8). Furthermore, the overall induction of *xCT* in PCa is confirmed by results from the database GEPIA (Fig 5B). In total, these findings indicate both an induction and an increase in xCT expression corresponding to higher Gleason scores.

Similar trends are obtained for *AL122023*.*1* expression in these samples. Comparing PCa with tumor-adjacent normal tissues, there was an average 1.7-fold increase (log2 ≈ 0.8 in ≥GS8, not significant) in *AL122023*.*1* expression with increasing Gleason scores (Fig 5A). These findings were further supported by GEPIA database results, confirming the induction of *AL122023*.*1* in PCa (Fig 5B).

Next, subsequent immunohistochemical staining of PCa tissue revealed the localization of xCT. For this purpose, four tissue microarrays (TMAs) were used containing a total of 196 prostate punch biopsies with different Gleason scores from 174 individual patients aged 47–78 years. When two samples per patient were available and they differed in Gleason score, both were included in the analysis, which was the case for 22 patients.

**Fig 6:**
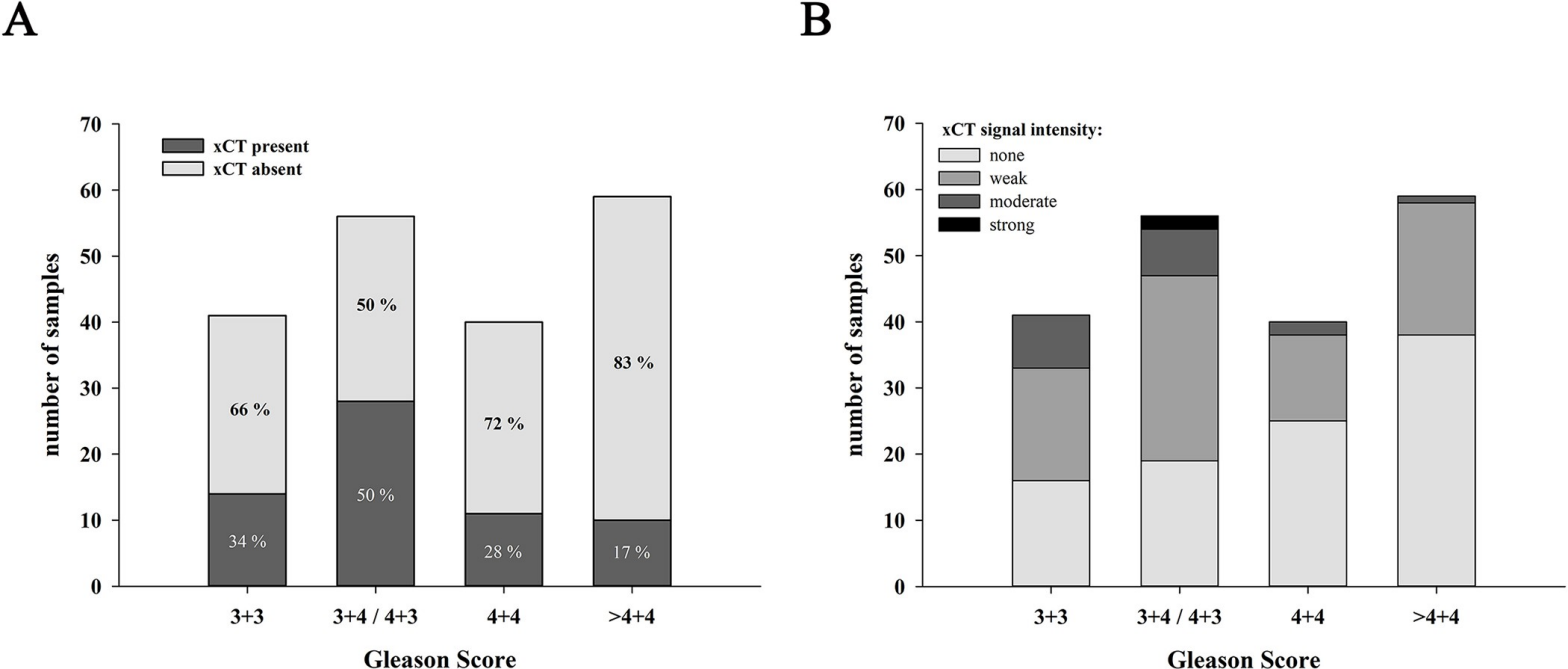
Presence and expression intensity of xCT in tumor cells or stroma depending on the Gleason score. (A) 196 PCa biopsies with different Gleason scores were assessed after immunohistochemical staining with anti-xCT-antibodies distinguishing between xCT-negative (no detection of tumor cells expressing xCT) and xCT-positive (detection of xCT expression in tumor cells) samples. Percentage values inside bars refer to the total number of each Gleason Score group. (B) The staining intensity of the adjacent stroma for xCT classified into absent, weak, moderate, or strong staining. (n = 3+3 = 41; 3+4/4+3 = 56; 4+4 = 40; >4+4 = 59).

First, the general presence or absence of xCT-positive cells was determined (Figs 5C and 6A). Cells with a precise xCT signal are detected in 63 samples, while 133 are negative. As already observed in healthy prostate tissue (fetal, juvenile, adult) and BPH, anti-xCT staining in PCa tissue is predominantly localized to individual cells in the glandular epithelium. In samples with a Gleason score of 6, only a few isolated cells were xCT-positive. This number of xCT-positive cells per sample as well as samples even displaying stained cells slightly increases for Gleason score 7. Conspicuously, from a Gleason score of 8 onward, a higher proportion of samples are xCT-negative. However, if xCT is detected, the number of xCT-positive cells within one sample increases significantly. Overall, there appears to be an accumulation of xCT-positive cells in some specific PCa samples with increasing Gleason score.

The presence of xCT was also examined in the adjacent stroma. For this purpose, the stroma was classified into four categories: no staining, weak, moderate, or strong staining as visually categorized in Fig 5D. The corresponding results for the number of samples per expression intensity are summarized in Fig 6B.

A similar expression pattern and intensity in the stroma is identified as seen for healthy prostate tissue and BPH. In general, either no or a very weak xCT expression is detected in stromal cells across all Gleason scores. Few samples display a moderate staining and only two samples with Gleason score 7 even showed a strong staining. Moreover, there is no correlation between the tumor-adjacent stroma and the presence or amount of tumor cells, whether xCT-positive or -negative.

**Fig 7:**
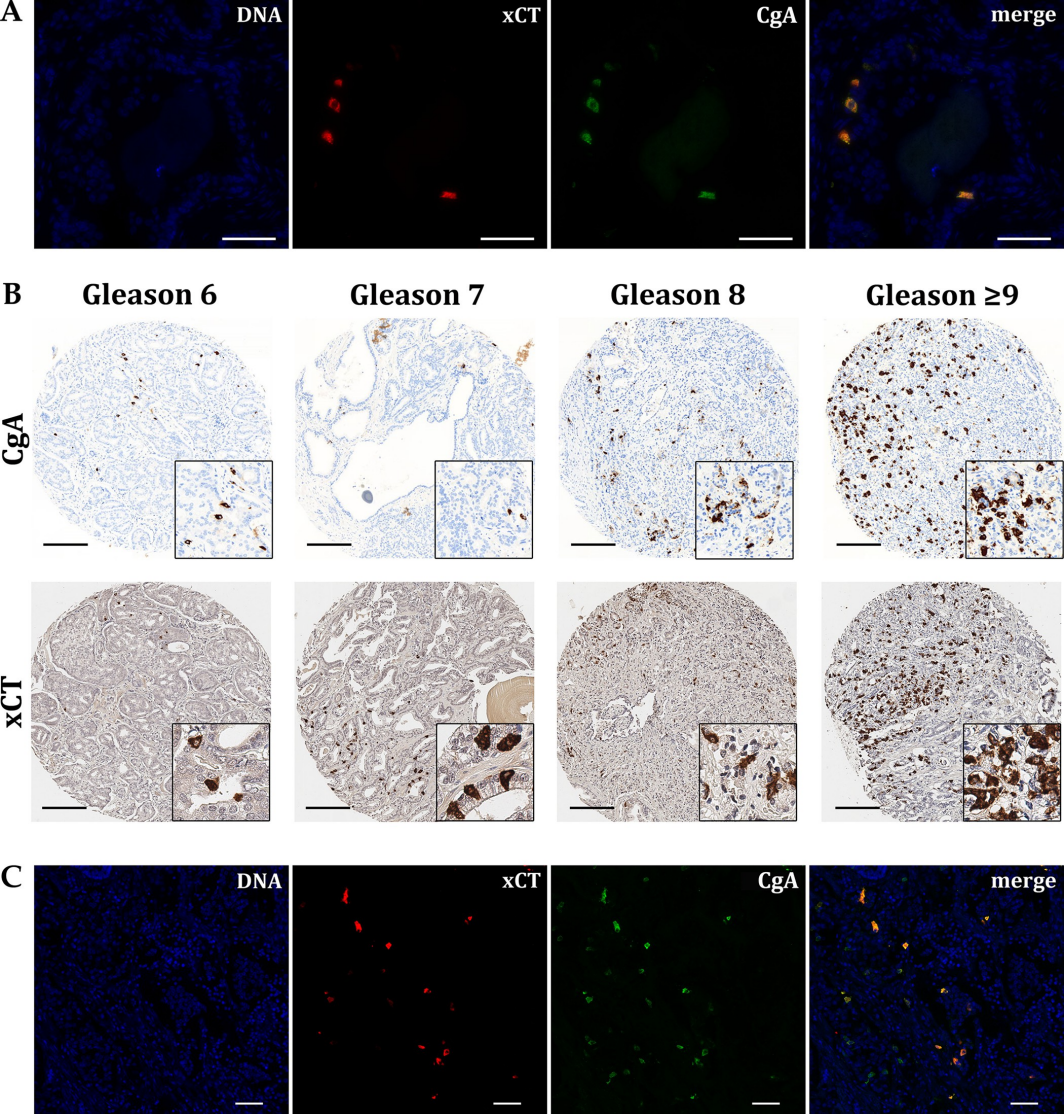
Examination of cells expressing xCT. (**A**) Double immunofluorescence staining of xCT (red) and CgA (green) in healthy prostate tissue was first performed showing a clear colocalization (merge). The fluorochrome DAPI visualizes DNA in the cell nuclei (blue). Scale bar, 50 μm. (**B**) Detection of xCT and CgA in a selection of PCa biopsies with different Gleason scores (column-wise) from a TMA. Comparison of CgA and xCT shows a similar staining pattern. Morphologic differences result from different sectioning levels. Scale bar, 200 μm. (**C**) To detect neuroendocrine-like tumor cells in PCa after androgen deprivation therapy, corresponding double immunofluorescence staining were performed (n = 5) confirming colocalization of xCT and CgA. Scale bar, 50 μm.

Since only single cells in the glandular epithelium repeatedly appeared xCT-positive in both healthy juvenile and adult prostate tissue as well as in BPH and partly in PCa tissue, we hypothesized that these represent neuroendocrine cells. Therefore, a healthy prostate tissue sample from a 64-year-old man, which showed comparatively many cells with distinct xCT expression, was selected for double-immunofluorescence staining for the neuroendocrine marker chromogranin A (CgA) (Fig 7A).

The double staining of xCT and CgA reveals an evident colocalization and therefore confirms the hypothesis that neuroendocrine cells in the glandular epithelium of the prostate express xCT. Next, it was investigated whether xCT-positive cells in PCa also represent neuroendocrine cells. For this purpose, TMAs stained with anti-xCT antibodies were compared to TMAs prior stained with anti-CgA antibody by the University of Bern (Fig 7B). Morphological differences in PCa sample result from different sectioning levels in the tissue cylinder. The optical differences are the result of different scanning methods. Again, the anti-xCT staining pattern closely resembles the anti-CgA staining, suggesting that cells co-express xCT and CgA. To finally verify whether neuroendocrine-like tumor cells in PCa also show the same expression pattern after androgen deprivation therapy, five rare samples were analyzed. A representative staining result is shown in Fig 7C. For neuroendocrine-differentiated prostate tumors, we demonstrate that xCT is exclusively detected in CgA-positive cells. In summary, these results show for the first time that neuroendocrine cells in healthy and pathological prostate tissue as well as transdifferentiated neuroendocrine-like cells express xCT.

## Discussion

We previously published results of a deregulated mRNA expression in LNCaP cells after interaction with stromal p21 cells via soluble factors [8]. Here, the repression of *xCT* and *AL122023*.*1* after seven days of co-culture was verified by qRT-PCR in three independent experiments. Moreover, validation of the repressed expression of *xCT* and *AL122023*.*1* after reverse co-cultivation argues for apical and basolateral secretion of soluble factors. Finally, Western Blot analysis also confirmed the repression of xCT at the protein level. Notably, there is an ongoing debate about the expected result in a western blot hybridization for xCT. Several public databases (NCBI, Uniprot) predict a molecular weight of around 55 kDa for the 501 amino acid protein. Indeed, Chen et al. [24] identified a xCT band at around 57 kDa, whereas Liefferinge and colleagues demonstrated that the actual size is closer to 35 kDa [25]. Thus, the characteristics of the antibodies used in this study was tested by overexpression of xCT in cells, protein extraction and subsequent analysis by Western Blotting (S3 Fig). xCT (1506 bp) cloned in pcDNA3.1 vector was obtained by Vectorbuilder and the sequence was double-checked by a commercial vendor (LGC Genomics, Berlin, Germany). Using three different antibodies (PA1-16893, Invitrogen; #711589 (clone 3HCLC), Invitrogen; 12691S (clone D2M7A), Cell Signaling), xCT was uniquely identified at the level of 35 kDa.

As the RNA sequencing data were repeatedly confirmed by different experiments, xCT and AL122023.1 were further investigated. Here, they were found to be linked by miR-26a, miR-30d and miR-30e. In this regard, Wach and colleagues, similar to our own results, showed a direct correlation between the induced expression of miR-26b and the corresponding induction of xCT in collecting duct renal cell carcinoma [26]. Studies on the interaction of miR-26b or -30b and xCT have already confirmed the functionality of the binding sites via luciferase reporter assays [27–29]. Since miR-26a, -30d and -30e belong to the same miRNA family, they share the same core seed sequence that is crucial for binding. Therefore, they are expected to bind to the 3’UTR of xCT and thus inhibit xCT expression. However, the results of our Western Blot analyses showed an average induction of xCT expression when either of the three miRNAs was ectopically expressed. According to the DIANA tool TarBase v.8, those miRNAs have a variety of potential target genes. Therefore, the elevated expression of xCT might be caused by repressing other target proteins and enhancing translation. Moreover, it is conceivable that only a combination of miRNAs could provide an inhibition of xCT expression in prostate cancer cells. In addition, lncRNAs are becoming increasingly important in terms of their miRNA regulatory function. For example, the lncRNA TUG1 has been shown to play a role in PCa development by regulating the function of miR-26a [30]. Congruently, this work identified the lncRNA AL122023.1 as a potential interacting partner for the miR-26 and miR-30 families. A repressed expression of AL122023.1 would result in an elevated concentration of miRNA molecules that, by a yet unknown pathway, could inhibit the expression of *xCT*. Since *xCT* is also repressed after seven days of co-culture in LNCaP cells, there might be a causal relationship. Luciferase reporter assays confirmed the unambiguous binding of miR-26a, -30d and -30e to the predicted binding sites within *AL122023*.*1*. Furthermore, we determined the indirect effect of AL122023.1 on the expression of xCT in LNCaP cells by Western Blot analysis. The results showed a corresponding increase in xCT expression by 15% on average. This relatively weak induction of xCT could either be due to a low endogenous miRNA level or due to the fact that lncRNAs only scavenge miRNAs for a certain period of time but do not degrade them. Thus, miRNAs could possibly lead to the inhibition of xCT expression again after a time delay. Corresponding kinetics would provide new insights. We also showed an induced *AL122023*.*1* expression in PCa with increasing Gleason score. Consequently, *xCT* expression also increased, again suggesting a causal relationship between AL122023.1 and xCT.

Immunohistochemical staining was intended to provide an overview of xCT expression in prostate tissue from various origins. During prostate development, only very few basally located cells within glandular epithelium express xCT. The stroma predominantly shows minimal or no xCT expression, although there are also marginal areas with clearly stained cells. These areas, however, are not further characterized. In general, the function of xCT during embryogenesis has been poorly investigated. xCT knockout mice (xCT-/-) of both sexes exhibited no lethality and normal fertility [31]. Only blood plasma showed an increased cystine concentration and a decreased intracellular GSH concentration [31]. Interestingly, comparable levels of intracellular cystine in embryonic fibroblasts was found in wild-type and knockout mice. These results suggest that cells have compensatory mechanisms to provide cystine and may also thus protect the cell from ferroptosis. In this context, transsulfuration of methionine or even ASC transporters (ASCT1/ SLC1A4, ASCT2/ SLC1A5) represent a crucial alternative [32]. Furthermore, an age-related expression change was investigated using healthy tissue of male differing in age (9 month to 84 years). Analysis revealed a distinct staining of individual cells in the glandular epithelium, which appeared to be neuroendocrine cells. The stroma, similar to fetal prostate tissue, was largely xCT-negative except for individual cells in specific areas. Overall, there was no age-related increase or decrease of xCT-positive cells in the glandular epithelium or in cells of the stroma. Additionally, there was a clear staining of adjacent skeletal muscle. This suggests that xCT-positive cells in the stroma can also be muscular cells. Conveniently, Huang and colleagues found that there is a relationship between sarcopenia, the age-related decrease in muscle mass and strength, and ferroptosis [33]. They attributed accumulation of iron in muscle to p53-related repression of xCT. Next, the investigation of xCT expression in benign prostatic hyperplasia (BPH) featured same characteristics as healthy tissue. Thus, it can be concluded that xCT does not play a specific role in BPH.

The correlation of xCT deregulation and PCa is currently gaining interest. Various therapeutics are already described for a targeted induction of ferroptosis in castration-resistant PCa. These include Flubendazole [34], Sulfasalazine [35,36], Sorafenib [37], Erastin, and RSL-3 [38]. Complementarily, we detected an average induction of xCT expression in PCa with increasing Gleason score at both the RNA and protein level. With increasing Gleason score, an accumulation of xCT-positive cells within a sample was more likely. However, xCT was detected in fewer samples overall above a Gleason score of 8. Additionally, patient-related data did not indicate corresponding factors, such as PSA concentration or therapeutic intervention. However, this study demonstrated that xCT-positive tumor cells were neuroendocrine or at least neuroendocrine-like cells as they also expressed the neuroendocrine marker protein chromogranin A [39,40]. In our previously published studies, the co-culture of P21 and LNCaP cells leading to xCT induction also resulted in an increase of the neuroendocrine markers NSE (500%) and CD56 (20%). Furthermore, androgen deprivation of LNCaP cells led to a neuroendocrine-like phenotype of prostate cancer cells with an induction of xCT expression by 50% [41]. Therefore, xCT might serve as potential marker protein for advanced PCa. Besides, these results also highlight the relevance for therapeutic interventions, as androgen-resistant tumor cells can apparently escape ferroptosis by increasing xCT expression. In the future, effective treatments for advanced PCa could be achieved by targeting ferroptosis in tumor cells. According to a recent study, the combination of the androgen receptor antagonist enzalutamide and the ferroptosis-inducing substances erastin or RSL3 has an inhibitory effect on prostate tumor growth in vivo [38]. Also, the parallel administration of sulfalazine together with the vasodilator oxyfedrine made tumor cells more susceptible to ferroptosis [42].

In conclusion, this work shows that androgen-sensitive prostate carcinoma cells exhibit reduced xCT expression in the presence of stromal cells *in vitro*, while xCT is increasingly expressed in androgen-resistant, neuroendocrine-like cells in PCa tissue. Accordingly, this work highlights the relevance of the tumor microenvironment and underscores the necessity of studying cell-cell communication to better understand the development and progression of PCa. Finally, xCT as an important regulator of ferroptosis generates numerous new research approaches associated with a high clinical relevance. xCT may serve as a novel neuroendocrine marker and is also progressively gaining importance as a therapeutic target in prostate cancer.

## Supporting information

S1 File (PDF)

S1 Fig Immunohistochemistry and immunofluorescence for the confirmation of antibody specificity. (**A**) Rabbit-anti-xCT antibody staining using rat stomach tissue as positive control. For negative or isotype controls, IgG fraction of non-immunized rabbits or mouse anti-rat-CEACAM1 (IgG κ) were used (both kindly provided by B. B. Singer). Scale bar, 200 μm. (**B**) Representative immunohistochemistry of fetal prostate tissue with anti-cytokeratin 7 antibody to detect glandular epithelium and anti-desmin antibody to identify muscle cells in the stroma. Scale bar, 1000 μm. (**C)** For mouse-anti-CgA antibody, human ileum served as positive tissue control. Scale bar, 100 μm.
(PDF)

S2 Fig Representative immunohistochemical staining with anti-xCT antibody. Healthy prostate tissue of different donor age (**A-C, E**) and BPH (**D,F**). The endothelium of small vessels appears weakly xCT-positive (**A-D**) while perikarya of neurons show a more distinct staining (**E-F**). Scale bar, 200 μm.
(PDF)

S3 Fig Representative Western Blots for anti-xCT antibody specifity and xCT expression in LNCaP-LNCaP co-culture. **(A)** Western Blot analyses of xCT antibody specificity with HEK293T cell lysates (1, control transfection with empty vector; 2, xCT overexpression) using different anti-xCT-antibodies (from left to right; 12691S (clone D2M7A), Cell Signaling; PA1-16893, Invitrogen; #711589 (clone 3HCLC), Invitrogen). xCT can be uniquely identified with a molecular weight of 35 kDa. GAPDH (36 kDa) served as loading control. mAB, monoclonal antibody; pAB, polyclonal antibody. (**B**) Exemplary blot of kinetics of xCT expression in control co-cultures with LNCaP cells only (n = 3). The expression fluctuated between stable and a moderate increase of xCT, indicating dynamic regulation under these conditions.
(PDF)

S4 Fig Verification of functional plasmids for pcDNA3.1-AL122023.1, pSG5-miR-26a, pSG5-miR-30d and pSG5-miR-30e on RNA level. (**A**) Validation of overexpression of miRNAs and AL122023.1 in LNCaP cells and HEK293T cells by quantitative real-time PCR. (**B**) Northern Blot results of miRNA overexpression in HEK293T cells. The empty effector plasmid pSG5 served as negative control. RNA was priorly visualized by GelRed and UV light to control the loading effectiveness.
(PDF)

S1 Table Oligonucleotide pairs and resulting fragment size for quantitative real-time PCR. For amplification of miRNAs as well as 5S rRNA (reference gene), the following oligonucleotide pairs were purchased from Qiagen (Hilden, Germany): hsa-miR-26a-5p (YP00206023), hsa-miR-30d-5p (YP00206047), hsa-miR-30e-5p (YP00204714), and 5S rRNA (YP00203906).
(PDF)

S2 Table 5’ biotinylated probes for Northern Blotting. (PDF)

S3 Table Oligonucleotide sequences for cloning. Oligonucleotide sequences with highlighted cut site for restriction endonucleases (underlined) or changed complementary sequence corresponding to the seed sequence (bold) for molecular cloning int o the target vector and resulting fragment size.
(PDF)
