# Supplementary figures and images for: xCT as a potential marker for neuroendocrine cells in high-risk prostate cancer and the relation to AL122023.1-miR-26a/30d/30e axis
Source: PLoS One. 2025 Jan 27;20(1):e0318213. doi: 10.1371/journal.pone.0318213 (PMC11771886; doi:10.1371/journal.pone.0318213)

Fig 1B

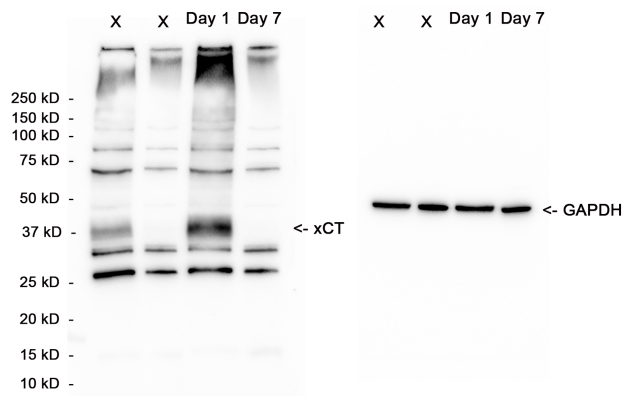

Fig 1C

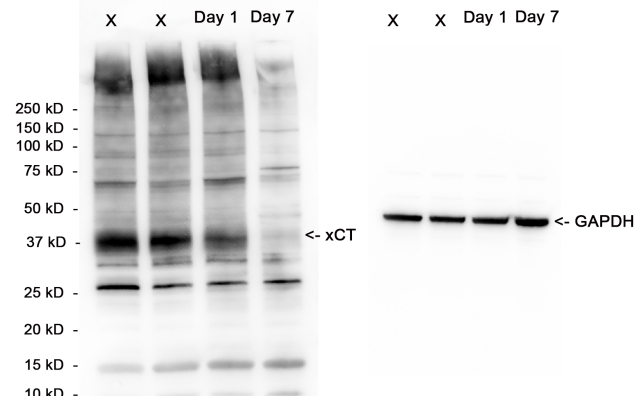

Fig 2D

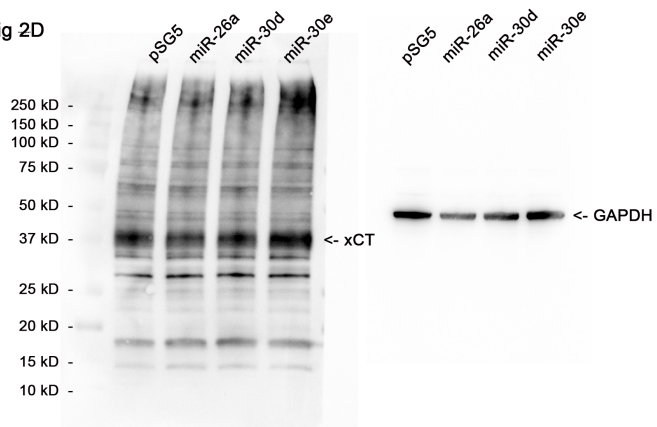

Fig 2E

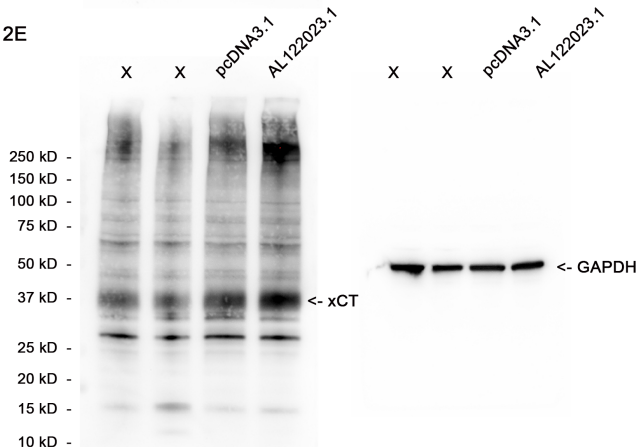

S3 Fig A

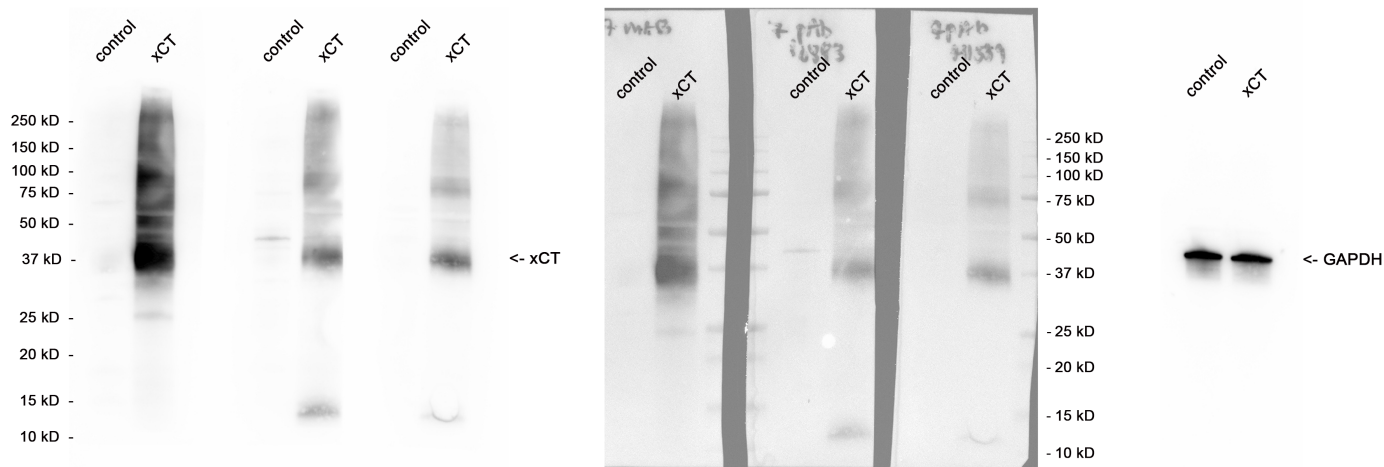

S3 Fig B

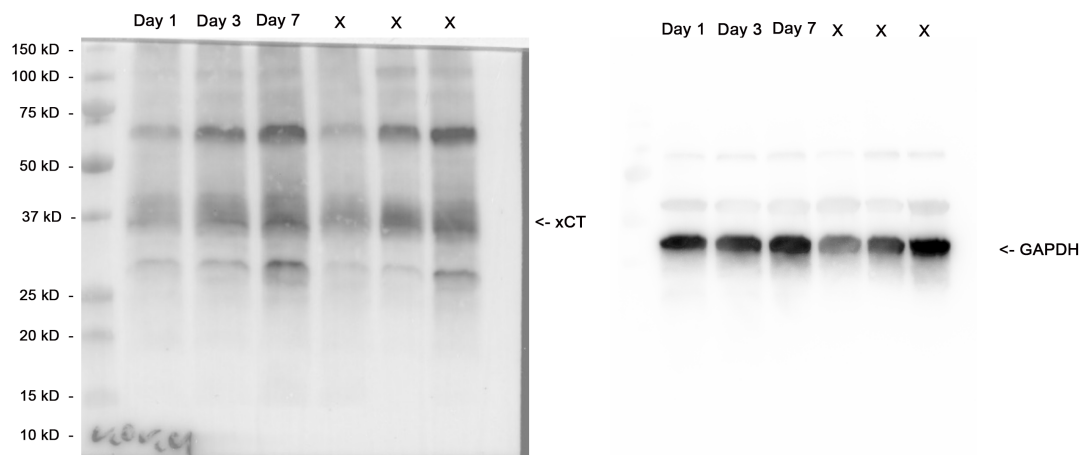

Supplement: S1 File — (PDF) [file pone.0318213.s001.pdf]

**S1 Fig.**

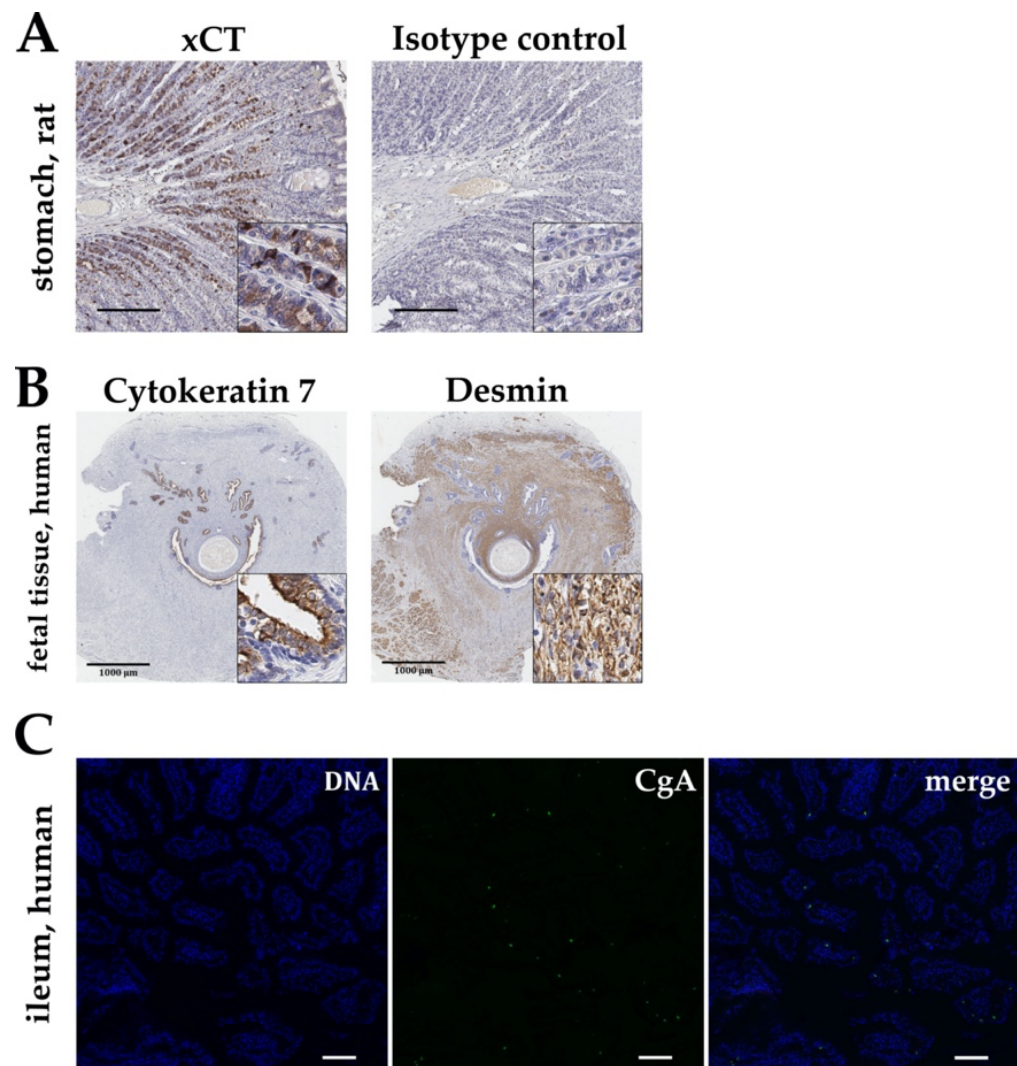

Supplement: S1 Fig — (A) Rabbit-anti-xCT antibody staining using rat stomach tissue as positive control. For negative or isotype controls, IgG fraction of non-immunized rabbits or mouse anti-rat-CEACAM1 (IgG κ) were used (both kindly provided by B. B. Singer). Scale bar, 200 μm. (B) Representative immunohistochemistry of fetal prostate tissue with anti-cytokeratin 7 antibody to detect glandular epithelium and anti-desmin antibody to identify muscle cells in the stroma. Scale bar, 1000 μm. (C) For mouse-anti-CgA antibody, human ileum served as positive tissue control. Scale bar, 100 μm. (PDF) [file pone.0318213.s002.pdf]

**S2 Fig.**

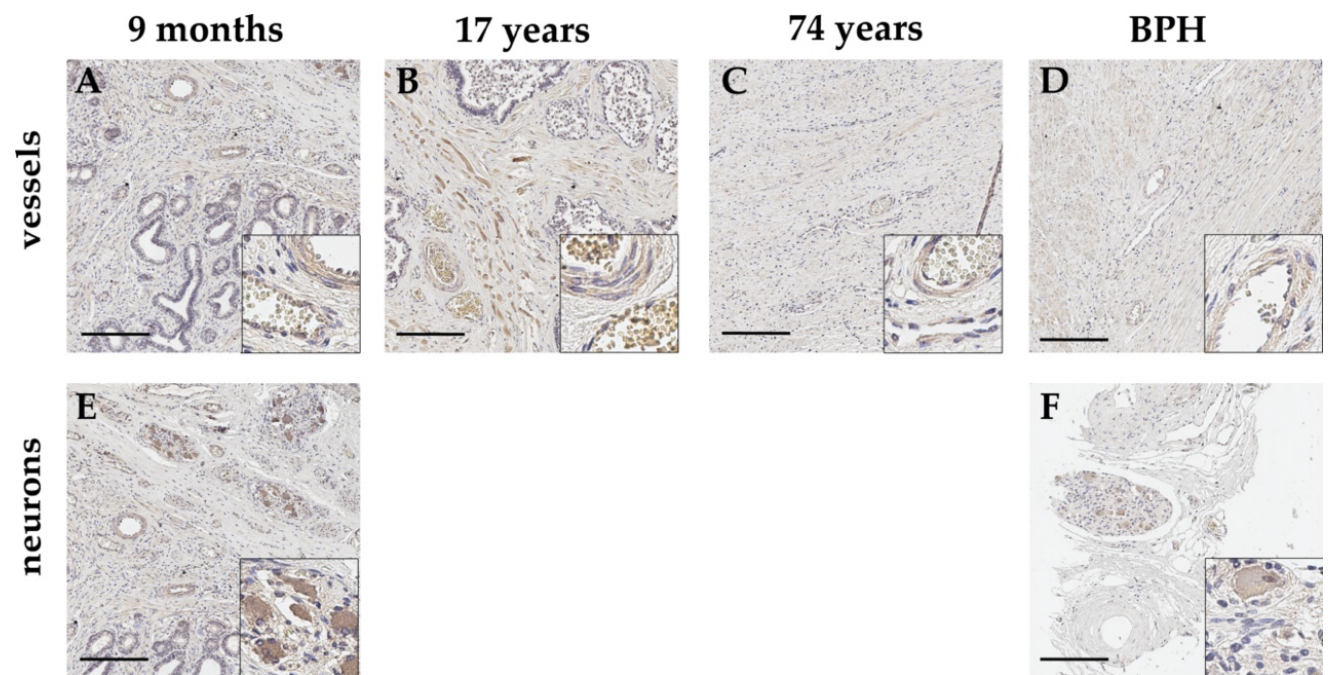

Supplement: S2 Fig — Healthy prostate tissue of different donor age (A-C, E) and BPH (D,F). The endothelium of small vessels appears weakly xCT-positive (A-D) while perikarya of neurons show a more distinct staining (E-F). Scale bar, 200 μm. (PDF) [file pone.0318213.s003.pdf]

### S3 Fig.

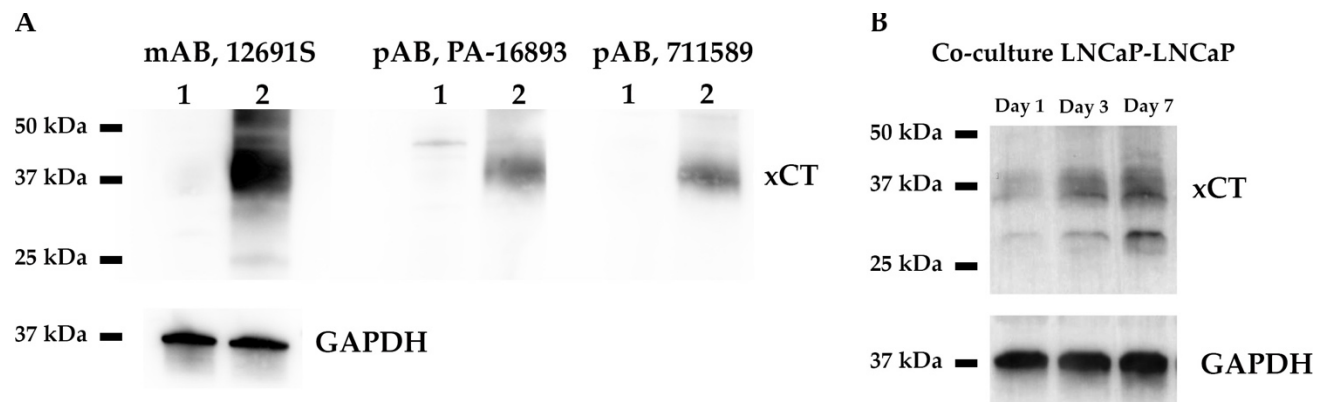

Supplement: S3 Fig — (A) Western Blot analyses of xCT antibody specificity with HEK293T cell lysates (1, control transfection with empty vector; 2, xCT overexpression) using different anti-xCT-antibodies (from left to right; 12691S (clone D2M7A), Cell Signaling; PA1-16893, Invitrogen; #711589 (clone 3HCLC), Invitrogen). xCT can be uniquely identified with a molecular weight of 35 kDa. GAPDH (36 kDa) served as loading control. mAB, monoclonal antibody; pAB, polyclonal antibody. (B) Exemplary blot of kinetics of xCT expression in control co-cultures with LNCaP cells only (n = 3). The expression fluctuated between stable and a moderate increase of xCT, indicating dynamic regulation under these conditions. (PDF) [file pone.0318213.s004.pdf]

S4 Fig.

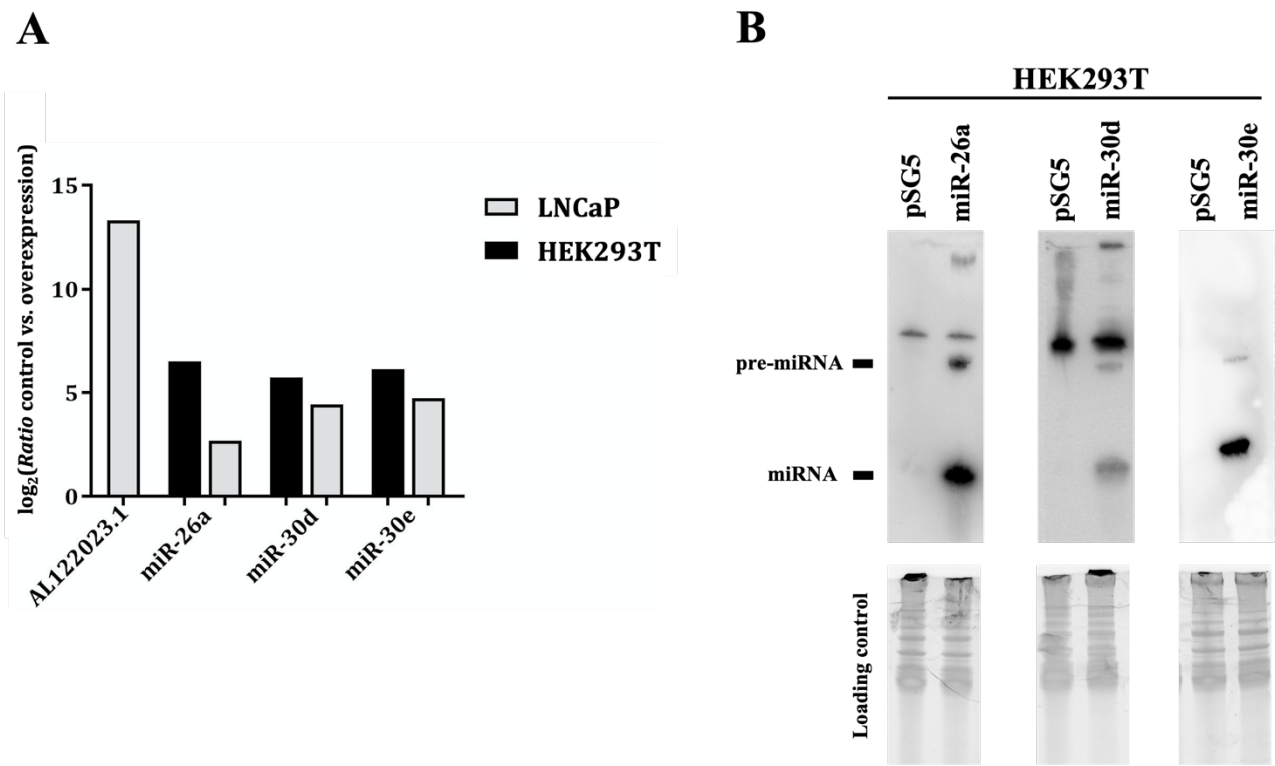

Supplement: S4 Fig — (A) Validation of overexpression of miRNAs and AL122023.1 in LNCaP cells and HEK293T cells by quantitative real-time PCR. (B) Northern Blot results of miRNA overexpression in HEK293T cells. The empty effector plasmid pSG5 served as negative control. RNA was priorly visualized by GelRed and UV light to control the loading effectiveness. (PDF) [file pone.0318213.s005.pdf]
