# Supplementary material for: xCT as a potential marker for neuroendocrine cells in high-risk prostate cancer and the relation to AL122023.1-miR-26a/30d/30e axis
Source: PLoS One. 2025 Jan 27;20(1):e0318213. doi: 10.1371/journal.pone.0318213 (PMC11771886; doi:10.1371/journal.pone.0318213)
Supplement: S1 Table — For amplification of miRNAs as well as 5S rRNA (reference gene), the following oligonucleotide pairs were purchased from Qiagen (Hilden, Germany): hsa-miR-26a-5p (YP00206023), hsa-miR-30d-5p (YP00206047), hsa-miR-30e-5p (YP00204714), and 5S rRNA (YP00203906). (PDF) [file pone.0318213.s006.pdf]

S1 Table.

Gene	Sequence (5′-3′), complementary reverse	Amplicon size (bp)
5′AL122023.1	CATGGGATTCTAAACACCGTATTG	83
3′AL122023.1	CATACTCAAGTCTGCCTGTCC	
5′ GAPDH	TGGAGAAGGCTGGGGCTCAT	176
3′ GAPDH	GACCTTGGCCAGGGGTGCTA	
5′ HPRT1	GCATACCTAATCATTATGCTGAGG	87
3′HPRT1	CTCGAGCAAGACGTTCAGTC	
5′ SLC7A11/xCT	TCATGTCCGCAAGCACACTCC	210
3′ SLC7A11/xCT	GGGATGAACAGTGGCACCTTGA	
