# Supplementary material for: xCT as a potential marker for neuroendocrine cells in high-risk prostate cancer and the relation to AL122023.1-miR-26a/30d/30e axis
Source: PLoS One. 2025 Jan 27;20(1):e0318213. doi: 10.1371/journal.pone.0318213 (PMC11771886; doi:10.1371/journal.pone.0318213)
Supplement: S2 Table — (PDF) [file pone.0318213.s007.pdf]

S2 Table.

miRNA	Sequence (5′-3′), antisense reverse
miR-26a-1-5p	AGCCUAUCCUGGAUUACUUGAA
miR-30d-5p	CUUCCAGUCGGGGAUGUUUACA
miR-30e-5p	CUUCCAGUCAAGGAUGUUUACA
