# Supplementary material for: xCT as a potential marker for neuroendocrine cells in high-risk prostate cancer and the relation to AL122023.1-miR-26a/30d/30e axis
Source: PLoS One. 2025 Jan 27;20(1):e0318213. doi: 10.1371/journal.pone.0318213 (PMC11771886; doi:10.1371/journal.pone.0318213)
Supplement: S3 Table — Oligonucleotide sequences with highlighted cut site for restriction endonucleases (underlined) or changed complementary sequence corresponding to the seed sequence (bold) for molecular cloning int o the target vector and resulting fragment size. (PDF) [file pone.0318213.s008.pdf]

S3 Table.

Vector	Gene and restriction endonuclease	Sequence (5′-3′)	Fragment size
pMIR	5′ AL122023.1_SpeI<br>3′ AL122023.1_SacI	cgACTAGTcagtgtccacctcttgttcc<br>cgGAGCTCcttctattgttgttgatacc	548
	5′AL122023.1_mut_26a<br>3′AL122023.1_mut_26a<br>5′AL122023.1_mut_30<br>3′AL122023.1_mut_30	ttactaacctttt**GCGCACCTG**ctttgcttctat<br>atagaagcaaag**CAGGTGCGC**aaaggttagtaa<br>gatcagtccccac**CAGCGGT**acttattactaac<br>gttagtaataagt**ACCGCTG**gtggggactgatc	530
pcDNA	5′ AL122023.1_HindIII<br>3′ AL122023.1_EcoRI	cgAAGCTTagtgtccacctcttgttcc<br>cgGAATTCttctattgttgttgatacc	546
pSG5	5′ miR-26a_EcoRI<br>3′ miR-26a_BamHI	cgGAATTCtgctgacccattcttgca<br>cgGGATCCccacaagactcctcgttgc	423
	5′ miR-30d_EcoRI<br>3′ miR-30d_BamHI	cgGAATTCtaggagaaattgcacttggtga<br>cgGGATCCcacgctaagtctgggaagc	466
	5′ miR-30e_EcoRI<br>3′ miR-30e_BamHI	cgGAATTCggagcaaagctgtgccttg<br>cgGGATCCggaactgacaggatgtgac	714
